# Bisphosphonate-Related Osteonecrosis of the Jaw and Oral Microbiome: Clinical Risk Factors, Pathophysiology and Treatment Options

**DOI:** 10.3390/ijms25158053

**Published:** 2024-07-24

**Authors:** Sapir Jelin-Uhlig, Markus Weigel, Benjamin Ott, Can Imirzalioglu, Hans-Peter Howaldt, Sebastian Böttger, Torsten Hain

**Affiliations:** 1 German Center for Infection Research (DZIF), Partner Site Giessen-Marburg-Langen, Justus Liebig University Giessen, D-35392 Giessen, Germany; sapir.jelin@med.uni-giessen.de (S.J.-U.); markus.weigel@mikrobio.med.uni-giessen.de (M.W.); can.imirzalioglu@mikrobio.med.uni-giessen.de (C.I.); 2 Institute of Medical Microbiology, Medical Microbiome-Metagenome Unit (M3U), Justus Liebig University Giessen, D-35392 Giessen, Germany; benjamin.ott@mikrobio.med.uni-giessen.de; 3 Department of Oral and Maxillofacial Surgery, Justus Liebig University Giessen, University Hospital Giessen and Marburg, D-35392 Giessen, Germany; hp.howaldt@uniklinikum-giessen.de (H.-P.H.); sebastian.boettger@uniklinikum-giessen.de (S.B.)

**Keywords:** bisphosphonate-related osteonecrosis of the jaw (BRONJ), antiresorptive drug-related osteonecrosis of the jaw (ARONJ), oral microbiome, pathophysiology

## Abstract

Bisphosphonate-related osteonecrosis of the jaw (BRONJ) represents a serious health condition, impacting the lives of many patients worldwide. The condition challenges clinical care due to its complex etiology and limited therapeutic options. A thorough understanding of the pathophysiological and patient-related factors that promote disease development is essential. Recently, the oral microbiome has been implicated as a potential driver and modulating factor of BRONJ by several studies. Modern genomic sequencing methods have provided a wealth of data on the microbial composition of BRONJ lesions; however, the role of individual species in the process of disease development remains elusive. A comprehensive PubMed search was conducted to identify relevant studies on the microbiome of BRONJ patients using the terms “microbiome”, “osteonecrosis of the jaws”, and “bisphosphonates”. Studies focusing on symptoms, epidemiology, pathophysiology, risk factors, and treatment options were included. The principal risk factors for BRONJ are tooth extraction, surgical procedures, and the administration of high doses of bisphosphonates. Importantly, the oral microbiome plays a significant role in the progression of the disease. Several studies have identified alterations of microbial composition in BRONJ lesions. However, there is no consensus regarding bacterial species that are associated with BRONJ across studies. The bacterial genera typically found include *Actinomyces*, *Fusobacterium*, and *Streptococcus*. It is postulated that these microbes contribute to the pathogenesis of BRONJ by promoting inflammation and disrupting normal bone remodeling processes. Current therapeutic approaches are disease-stage-specific and the necessity for more effective treatment strategies remains. This review examines the potential causes of and therapeutic approaches to BRONJ, highlighting the link between microbial colonization and BRONJ development. Future research should seek to more thoroughly investigate the interactions between bisphosphonates, the oral microbiome, and the immune system in order to develop targeted therapies.

## 1. Introduction

Bisphosphonates (BPs) are antiresorptive drugs that directly inhibit osteoclast activity and thereby suppress bone resorption [1]. They play an important role in the treatment of diseases such as osteoporosis and bone metastases. However, the effectiveness of a treatment depends on the route of administration, the treatment’s concentration, and frequency of use [2]. One of the most serious adverse effects of BPs is bisphosphonate-related osteoporosis of the jaw (BRONJ). The diagnosis of BRONJ requires that patients meet three main criteria: (a) current or previous use of BPs; (b) the present of persistent exposed necrotic bone in the maxillofacial region that does not heal within eight weeks of diagnosis; and (c) an absence of previous radiation therapy of the head and neck [3].

BRONJ’s clinical manifestations cover a vast spectrum, from asymptomatic cases to severe pain, the swelling of soft tissues, infections, and sensory disturbances like paresthesia. Further complications can include tooth loss, the formation of both intra- and extraoral fistulae, oroantral fistulae, and even fractures of the jaw [4,5,6]. These side effects may severely impact a patient’s overall well-being and quality of life by causing pain and difficulty in eating and by worsening oral hygiene [7].

Importantly, BRONJ can be caused by drug classes other than BP, such as receptor activators of NF-κB ligand (RANKL) inhibitors like denosumab; hence, we use the new term antiresorptive drug–related osteonecrosis of the jaw (ARONJ). RANKL plays an important role in bone remodeling. It is a cytokine that is expressed in many cells, including osteoblasts, bone marrow stromal cells, and immune cells. It is crucial for the function, activation, and differentiation of osteoclasts, thereby reducing bone turnover. However, denosumab has several advantages over BPs, including better tolerability, ease of subcutaneous injection, a lower incidence of nephrotoxicity, and a shorter half-life [8].

The incidence rates of BRONJ vary considerably depending on the drug class, dosage, and the route of administration, ranging from 0.4% to 21% [3]. In oncological applications, BPs are typically administered at higher dosages and with greater frequency compared to other use cases like osteoporosis [9]. A recent study compared the incidence of BRONJ to the dosage of zoledronate by querying the French National Pharmacovigilance Database. The Reporting Odds Ratio (ROR) of BRONJ for the dosages used in oncology (4 mg/month) compared to those used in rheumatology (5 mg/year) was 16.40 [12.53–21.46], suggesting a significant increase in BRONJ risk with increasing zoledronate dosage [10]. Various studies have tried to shed light on the multifactorial etiology of BRONJ, with dental extractions and the intravenous application of BPs emerging as significant risk factors [11]. Spontaneous cases of BRONJ have also been reported [12,13]. However, the delineation between trauma-induced and spontaneous lesions is blurred by the oral cavity’s delicate mucosa, which is susceptible to injury, even during routine interventions [12]. The question of why BPs may increase the incidence of osteonecrosis of the jaw (ONJ) is the subject of intense debate. Studies suggest various underlying mechanisms, including disrupted bone remodeling, inflammation and oral microbial infection, immune system suppression, soft tissue toxicity, and the inhibition of angiogenesis [14,15]. Due to the unknown nature of the pathophysiological processes underlying BRONJ, effective therapeutic measures are currently lacking.

In exploring the relationship between oral health and systemic bone conditions, studies have focused on the unique physiological characteristics of the oral cavity, including its distinct blood supply [12], its specialized bone structure [9,12], and a diverse microbiota comprising over 750 bacterial species [3,16,17,18]. Metagenomic studies reveal a significant shift in the bacterial landscape of BRONJ, suggesting a link between altered microbial diversity and disease progression. Furthermore, emerging evidence highlights the role of microbial colonization, both bacterial and fungal, with the high prevalence of Actinomyces [19,20,21,22,23,24,25,26,27] being a critical factor in the development of BRONJ. This contrasts with other regions of the skeletal system, where BRONJ incidence is notably lower, emphasizing the unique susceptibility of the oral cavity to this disease [2].

In this review, we set out to discuss the existing literature on BRONJ, with a focus on the role of microorganisms in its pathogenesis. A nuanced understanding of the specific microbiota and their role in the disease process could potentially improve preventive strategies and therapeutic interventions for patients suffering from BRONJ.

## 2. Methodology

A comprehensive search was conducted in PubMed (U.S. National Library of Medicine) from June 2023 to February 2024, with the objective of identifying the relevant literature on the microbiome in patients with BRONJ. The search terms “microbiome”, “osteonecrosis of the jaws”, and “bisphosphonates” were used to guide the search. Without temporal limitations, a total of 26 results were identified. Only articles published in English were included in the review. We excluded articles that did not address the microbiome or that did not provide substantial information on the specified focus areas of symptoms, epidemiology, pathophysiology, risk factors, and treatment options of BRONJ.

## 3. Clinical Risk Factors of BRONJ

Clinical risk factors for the development of BRONJ encompass a range of patient- and treatment-related features. Among dental risk factors, tooth extraction was found to be the most prominent risk, followed by periodontal disease [28]. Pre-existing oral infectious or inflammatory conditions, including periapical infections, also elevate the risk of BRONJ. By modifying osteoclast function and quantity, these infections aggravate the risk of the disease, even in the absence of tooth extraction [29].

Moreover, the absorbed dose of BP medications constitutes a crucial risk factor. Prolonged treatment periods increase the likelihood of developing BRONJ significantly [5,30,31,32]. Notably, patients receiving monthly treatments for metastatic osteolytic disease are at the highest risk, especially following tooth extraction and dentoalveolar surgery [5,33]. Furthermore, the use of nitrogen-containing bisphosphonates (N-BPs), administered intravenously, is associated with a higher incidence of the disease [15].

The aforementioned factors lead to an increased susceptibility to BRONJ in cancer patients, who typically require more potent and more frequent administration of BP therapy over extended periods. In addition, concomitant treatment with corticosteroids, antineoplastic agents, and methotrexate (immunosuppressant) has also been shown to increase the risk of BRONJ. However, further research is needed to elucidate the underlying pathophysiology. With regard to systemic factors, it has been demonstrated that lifestyle habits such as tobacco use and alcohol abuse are associated with an increased incidence of BRONJ [14,15].

Furthermore, the demographic factors associated with BRONJ have been identified, including age and gender. The condition is most commonly found in individuals aged between 50 and 70, with a slight predominance in female patients [11,16,34,35]. Moreover, the mandible is more frequently affected by BRONJ than the maxilla [11,35]. These results imply that BRONJ has a multifactorial etiology, in which drug dosage, patient demographics, and local factors in the oral cavity all play a role in the development and progression of the disease.

## 4. Pathophysiology

**Figure 1:**
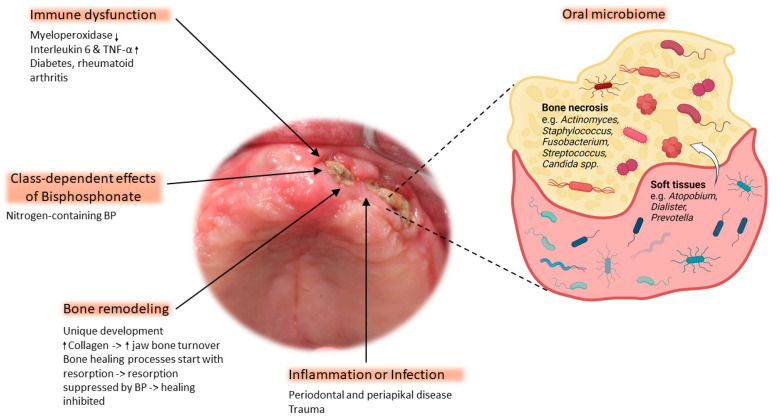
The pathophysiology of osteonecrosis of the jaw is based on five main factors: immune dysfunction, class-dependent effects of bisphosphonate (BP), bone remodeling, inflammation/infection, and the oral microbiome. The magnified area depicts the most abundant bacteria of the microbial composition commonly found within necrotic bone. The white arrow indicates bacterial migration from soft tissue to the exposed bone ↓ = decreased expression, ↑ = increased expression. Clinical image shows exposed bone with a necrotic lesion located on the left alveolar ridge of the edentulous maxilla (figure created with BioRender).

Despite considerable research efforts, the pathophysiology of BRONJ is still not fully understood, making effective treatment challenging. This section reviews the pathophysiological mechanisms underlying BRONJ (Figure 1) that are currently the most popular.

### 4.1. Immune Dysfunction

Recent research into BRONJ has revealed a possible connection to drug-induced immune dysfunction, leading to increased susceptibility to oral infections [36]. This new perspective challenges the previous belief that the oral microbiome directly causes BRONJ. Other factors, such as systemic diseases like rheumatoid arthritis and diabetes mellitus, may affect immune resilience and the body’s ability to respond to infection and inflammation [9]. It is hypothesized that people with BRONJ may have reduced immune resistance, which may affect their ability to cope with the immunological stress caused by N-BP [37]. Support for this theory comes from various molecular studies, such as PCR array analyses revealing notable upregulation in 34 genes and downregulation in 11 genes connected to inflammation in BRONJ samples [34]. In addition, tissue levels of myeloperoxidase, which regulates inflammatory processes, are significantly reduced in patients with BRONJ, and there is an increase in interleukin-6 (IL-6) and tumor necrosis factor-alpha (TNF-α). Genomic analysis also shows the downregulation of key genes involved in the antibacterial response and upregulation of genes involved in the immune response [5,34]. Furthermore, BPs have anti-angiogenic properties. They inhibit the proliferation, adhesion, and migration of human endothelial cells and suppress angiogenesis [38,39,40]. These findings emphasize the multifactorial pathophysiology of BRONJ, triggered by immune dysfunction, altered gene expression, and anti-angiogenic effects.

### 4.2. Class-Dependent Effects of Bisphosphonates on BRONJ Development

The class and administration of BP impact therapeutic results and related risks, especially in the context of BRONJ. BPs are classified into two categories: non-nitrogen-containing BPs (non-N-BPs) and N-BPs. Non-N-BPs, such as etidronate, are considered first-generation BPs, while N-BPs are second-generation BPs. This group includes, for example, alendronate and zoledronic acid. The newer group of BPs has distinct clinical applications and side effects and a high affinity for bone tissue. It inhibits the activity of farnesyl pyrophosphate synthase (FPPS), causing osteoclast apoptosis [2,41]. Zoledronic acid is the most potent BP and was the inaugural pharmaceutical agent to be approved for use in all solid tumors with bone metastases, including breast cancer, prostate cancer, multiple myeloma, and lung cancer [8]. In contrast, etidronate has lower affinity and accumulation, leading to a reduced inflammatory stimulus [42].

Microbial surface components recognizing adhesive matrix molecules (MSCRAMMs) are adhesive proteins known to play a critical role in facilitating the initial binding of Gram-positive bacteria to host tissues, a key step in the development of infection. The cationic nitrogen-rich domain of BPs is thought to interact with the amino-terminal regions of MSCRAMMs. This interaction is hypothesized to be a crucial element in the pathogenesis of BRONJ and might explain why the disease is less commonly seen with non-nitrogen-containing BPs [43,44]. In addition, N-BP disturbs the mevalonate pathway, which is essential for cholesterol and isoprenoid lipid synthesis. This leads to the suppression of protein prenylation, causing osteoclast apoptosis [45].

Further research into the complex interplay between the chemical structure of BPs, their binding properties, and bacterial interactions is key to understanding the pathogenesis of BRONJ.

### 4.3. Bone Remodeling

The suppression of bone remodeling by BPs is considered to be one of the key factors that causes BRONJ [46]. The impact of BPs on bone remodeling, together with the different developmental mechanisms of jaw bones compared to long bones, may explain the predominant occurrence of BRONJ in the jaw. While maxilla and mandible are formed via intramembranous bone development, long bones undergo endochondral ossification. This difference in developmental pathways leads to significant anatomical differences, including divergence in bone density and the balance between cortical and cancellous bone and marrow spaces. Interestingly, the human mandible has a higher collagen content and lower hydroxylysine levels than long bones, which could contribute to a higher rate of jaw bone turnover [47,48,49]. Importantly, the incorporation of BPs into bone is directly linked to the local bone turnover rate [50]. The differing bone healing processes that occur at different skeletal sites, affected by zoledronate, may constitute another explanation for the site-specific occurrence of BRONJ. For example, in the case of extraction sockets, healing typically begins with the resorption phase, whereas bone formation immediately sets in with tibial defects [20,51]. Zoledronate strongly suppresses bone resorption, thereby significantly inhibiting healing in extraction sockets but does not have a comparable effect on bone regeneration in tibial defects [26,42,47].

### 4.4. Inflammation and Infection

Common triggers for BRONJ include dental procedures, periodontal or periapical infections, trauma, or the use of poorly fitting prosthetic appliances [2,9,14,28]. These factors facilitate bacterial invasion and infection, which are particularly threatening in the context of the compromised bone health caused by BPs [9]. Several studies have indicated that patients with periapical and periodontal infections may be at an increased risk of developing BRONJ, regardless of whether or not they have undergone tooth extraction. This is thought to be due to the altered number and function of osteoclasts caused by the presence of infection [52]. Interestingly, some animal studies suggest that bone necrosis may occur before visible bone exposure, indicating the spontaneous development of BRONJ [5]. However, it is difficult to determine whether these lesions are caused by trauma due to the fragile mucosal lining of the oral cavity [12].

### 4.5. Oral Microbiome and BRONJ

The oral microbiome significantly contributes to the development and progression of BRONJ. The oral cavity harbors a diverse set of bacteria and yeast, making it an ideal environment for colonization and biofilm-related disorders [5]. Biofilms are structured communities of microorganisms that reside within self-produced extracellular polymeric substance (EPS) matrixes. These communities adhere to both living and non-living surfaces, exhibiting differences in growth rate and gene expression compared to their free-floating state. Biofilms allow microorganisms to establish relationships with the host, resist hostile external conditions, and withstand antibiotics and other environmental challenges by forming a protective barrier around themselves [53].

Furthermore, an acidic environment might be a key element in BRONJ’s pathogenesis. This environment is thought to be created by specific bacteria such as *Streptococcus* and other saccharolytic strains and further promoted by dental infections, invasive procedures, and the influence of N-BP [5,54]. However, the precise role of these bacteria, either as initiators or opportunistic colonizers of BRONJ lesions, is still under investigation [5]. Another crucial component of pathophysiology is the involvement of Gram-negative bacteria. These bacteria are believed to have a significant impact on the disease by promoting the differentiation and activity of osteoclasts [2].

In addition, treatment with BPs can lead to increased bacterial adhesion to bone surfaces, which may change the local microbiome and create an environment that promotes osteonecrosis. These observations emphasize the bidirectional relationship between the oral microbiome and BRONJ lesions [55,56].

The bacterial profiles obtained from patients with BRONJ exhibit clear differences compared to those observed in typical jawbone infections, such as dental caries and periodontal disease. The BRONJ phylotypes are not commonly associated with other infections in the jawbone, but they are known to cause other opportunistic infections [43,57]. Sedghizadeh et al. utilized scanning electron microscopy to examine biofilms in bone samples obtained from four patients with BRONJ who underwent surgical debridement [19]. The samples exhibited abundant biofilms, predominantly consisting of bacteria but also sporadically yeast, embedded in extracellular polymeric substances.

Although histological evaluations have detected a range of microorganisms on uncovered bone tissue, the specific oral bacteria responsible for BRONJ remain unknown. Typically, BRONJ lesions display a greater variety of microbial morphotypes compared to non-bisphosphonate-related osteomyelitis, which usually shows the dominance of a small number of different bacterial species such as *Actinomyces* sp., which is often the most prevalent species in BRONJ lesions [2]. A comprehensive overview of bacterial species linked to BRONJ is given in Table 1.

*Actinomyces* are commonly present in biofilms found at BRONJ sites, as corroborated by multiple studies, indicating a connection between these bacteria and the disease [19,21,22,23,24,25]. In addition, comparative shotgun metagenomic analyses have revealed an increase in the genus *Actinomyces* in patients with BRONJ in comparison to healthy individuals. This is accompanied by a decrease in *Streptococcus*, which is known to be the predominant microorganism in the oral microbiota of humans [7]. A recent study compared the microbial composition of BRONJ sites to non-infected sides by means of 16S metagenomic analysis of samples obtained from oral swabs. BRONJ sites showed the dominance of *Prevotella*, *Porphyromonas* and *Pyramidobacter* genera, while on the species level *Dialister pneumosinetes*, *Dialister ivisus*, and *Pseudoramibacter alatolyticus* were more abundant [3].

Also, *Fusobacteria* have been recognized as dominant in BRONJ [34]. This is supported by studies that demonstrate how infections caused by *Fusobacterium nucleatum* in the extraction sockets of mice following treatment with high-dose BP result in a delay in the healing of wounds, leading to the exposure of bone [58].

*Atopobium* sp. *oral taxon 199*, which is commonly found in the mucosal tissues of patients treated with BPs, was also observed in bone samples of BRONJ [16]. Moreover, bacterial genera such as *Dialister*, *Prevotella*, and *Atopobium*, which are typically found in soft tissues, are found in necrotic bone, indicating that they migrate from superficial oral sites to deeper bone tissues [16,25]. This supports the hypothesis that specific oral bacteria, which are typically not present in bone, can attach to and establish themselves in this habitat. This process may be worsened by the accumulation of BPs in the jawbone [59].

When studying the role of the microbiome in BRONJ, a crucial question emerges: which type of bacteria, Gram-positive or Gram-negative, has a greater impact on its development? Comprehending this differentiation is essential, as it has the potential to illuminate the development of BRONJ and direct efficacious treatment approaches.

In a study conducted by Pushalkar et al., the participants chosen for 16S rRNA sequencing were not administered antibiotics for approximately three months prior to sample collection in order to reduce the influence of antibiotics on bacterial colonization. The study unveiled a wide range of bacteria in the BRONJ cohort, for instance Gram-positive genera such as *Parvimonas*, *Eubacterium*, *Gemella*, *Leptotrichia*, and *Selenomonas*. Notably, the genera *Xanthomonas*, *Lachnospiraceae*, and *Bifidobacterium* were found only in the BRONJ group, suggesting a dysbiosis between Gram-positive and Gram-negative bacteria in the studied groups [34].

BRONJ samples frequently contain Gram-positive bacteria such as *Staphylococcus* and *Streptococcus* species, including *Staphylococcus pasteuri*, *Streptococcus parasanguinis*, *Streptococcus mitis*, *Streptococcus gordonii*, and *Streptococcus oralis*. The experimental groups treated with BPs exhibited increased hybridization signals in the biofilm present on teeth for these species [60]. Contrary to this, additional studies have failed to provide any evidence of *Staphylococcus aureus* in BRONJ lesions, indicating that other types of bacteria in the mouth are responsible for causing the disease [16].

Conversely, the participation of Gram-negative bacteria in the formation of BRONJ is a significant subject of interest. The presence of long-lasting inflammation in BRONJ, which is caused by infection, is frequently connected to Gram-negative bacteria and their byproducts, such as lipopolysaccharides. These factors are believed to play an important role in triggering the differentiation and activity of osteoclasts, which is a key process in the development of BRONJ [2,61].

Studies have indicated that necrotic bone contains a wide range of microorganisms, with a considerable abundance of Gram-negative bacteria observed in the majority of patients with BRONJ. A retrospective single-center study examined 116 bone samples from 98 patients. The analysis revealed that around 70% of the patients had bacteria that were resistant to β-lactamase inhibitors. The homogenized samples were cultured using conventional methodologies [35]. It is important to note that, in this study, patients who were diagnosed with stage 2 or stage 3 BRONJ were given a 7-day preoperative treatment of oral decontamination using chlorhexidine rinses and antibiotics such as amoxicillin with clavulanic acid, clindamycin, or moxifloxacin, depending on their tolerance to penicillin. The administration of this treatment may have caused a systemic bias in the recorded microbiome results.

Several studies have also demonstrated the existence of fungi, specifically yeast, in samples collected from patients with BRONJ. In the aforementioned retrospective study, yeast was identified in 21.5% of the samples [35]. Further, Jabbour et al. conducted DNA hybridization studies and discovered the presence of five Candida species in biofilm samples taken from both exposed bone and adjacent teeth [60]. This evidence indicates the necessity for a deeper understanding of the microbial dynamics in BRONJ, encompassing the influence of fungal infections in addition to bacterial involvement in disease.

According to a prevalent hypothesis [19,25,62,63], bone maintains its health until it sustains an injury and becomes infected with particular oral bacteria.

**Table 1:** Studies on microbes associated with bisphosphonate-related osteonecrosis of the jaw (BRONJ).

Microorganisms	Method	Reference
***Fusobacterium*, *Bacillus*, *Actinomyces*, *Staphylococcus*, *Streptococcus*, *Selenomonas*, *Treponemes*, *Candida* spp.**	Clinical study, histopathology from bone samples (H&E and SEM)	Sedghizadeh et al. (2008) [19]
***Prevotella*, *Porphyromonas* and *Pyramidobacter*. At the species level: *Dialister pneumosinetes*, *Dialister ivisus*, and *Pseudoramibacter alatolyticus*.**	Clinical study, split-mouth design, 16S metagenomic analysis from oral swab samples	Kim et al. (2024) [3]
***Actinomyces* spp.**	Retrospective study, histopathology	Abu-Id et al. (2008) [22]
***Actinomyces* spp.**	Clinical study, histopathology from bone tissue and skin-exhibiting fistula (H&E, PAS, gram, Grocott)	Hansen et al. (2006) [23]
***Actinomyces* spp.**	Retrospective study, histopathology (H&E, gram, PAS)	Kaplan et al. (2009) [24]
***Actinomyces* spp.**	Clinical study, shotgun metagenome sequencing from saliva samples	Yahara et al. (2020) [7]
***S. constellatus*, *Bifidobacterium dentium*, *Eubacterium infirnum*, *Selenomonas sputigena* and *uncultivable phylotypes*, *Actinomyces* sp. *oral taxon 525* and *Lachnospiraceae* sp. *oral taxon 086***	Clinical study, 16S rRNA cloning and sequence analysis, antibacterial response using ELISA, soft tissue samples associated with the necrotic bone	Pushalkar et al. (2014) [34]
***Streptococcus*, *Eubacterium*, *Pseudoramibacter***	Clinical study, culture-independent 16S rRNA gene-based molecular techniques from infected bone samples	X Wei et al. (2012) [16]
***S. pasteuri*** **, *S. parasanguinis*, *S. mitis***	in vivo study (mice), DNA checkerboard hybridization, samples of biofilm from sites of exposed bone and the supragingival region of the adjacent teeth	Jabbour et al. (2016) [60]
***Streptococcus* spp., *Neisseria* spp., *Lactobacillus* spp., Coagulase-negative Staphylococci, *Candida* spp.**	Retrospective study, Culture-dependent technique from bone samples	Ewald et al. (2020) [35]

H&E, hematoxylin and eosin; SEM, scanning electron microscopy; PAS, periodic acid—Schiff; ELISA, enzyme-linked immunosorbent assay.

The combination of this infection, along with the bone resorption reduction caused by BPs and the potential impairment of blood vessels, may impede the process of forming new bone, thus promoting the development of BRONJ [62,63,64]. This suggests that bacterial colonization in BRONJ may originate from the oral microbiome and be influenced by BP treatment and subsequent changes in oral and bone health. The issue of whether the oral microbiome plays a causal role in BRONJ is complex and has multiple aspects. Several studies indicate that the oral microbiome is not believed to be a direct cause of BRONJ. Individuals treated with N-BP typically have a deficiency in immune function due to their underlying disease. Therefore, the additional immune stress caused by N-BP treatments may put them at increased risk of developing BRONJ [37].

Conversely, other studies indicate that the bacteria associated with bone infections related to BRONJ may be distinct from those found in other biofilm-related bone infections in the oral cavity [19,25]. This includes a variety of microorganisms found in BRONJ lesions that are accountable for a range of opportunistic infections affecting the bones, joints, and teeth [16,60].

In summary, our current understanding points towards a multifactorial etiology for BRONJ, where the oral microbiome plays an important role but is not solely causal. Rather, it interacts with other factors such as immune responses and BP treatment, resulting in the emergence of BRONJ.

## 5. Treatment Options

Various treatment strategies have been developed for BRONJ. These depend on the stage and severity of the disease. Conservative approaches are recommended for early-stage BRONJ (stage 1). This includes patients who are asymptomatic and show no signs of infection. Only exposed and necrotic bone or fistulae penetrating the bone should be considered for these treatments [6]. These approaches include antibacterial mouth rinses, patient education, and routine monitoring [5]. A possible wound care protocol uses mouth rinses containing antimicrobial agents, such as 0.12% chlorhexidine and hydrogen peroxide [65,66,67,68,69]. These rinses aid in reducing bacterial colonization, with chlorhexidine being particularly effective against *Streptococcus mutans* and *Lactobacillus* [70]. Besides, chlorhexidine has antifungal properties and is effective against *Candida albicans*, a common yeast in BRONJ lesions [71].

BRONJ stage 2 patients suffer from exposed bone or fistulae that penetrate the bone and may develop infections, characterized by pain, redness, and purulent discharge [6]. Treatment for stage 2 involves intensified systemic antibiotics, pain management, and possibly the debridement of necrotic bone. It has been shown that systemic antibiotics alone are insufficient for preventing bacterial colonization and healing BRONJ lesions [5]. Research indicates that extended preoperative antibiotic therapy can considerably enhance healing outcomes. The success rate for complete healing was observed to be between 70% and 87%, while short-term treatment had a success rate of only 35% to 53% [72]. These figures highlight the potential involvement of bacteria in the progression of BRONJ. Postoperative antibiotic treatment should be continued until signs of local inflammation or infection have subsided, with follow-up visits undertaken to monitor and adjust therapy as necessary [35].

When treating BRONJ patients, it is crucial to consider the duration of antibiotic therapy, which can range from several months to more than a year. If lesions do not respond to oral treatment, intravenous antibiotics are a viable option [73,74].

It is worth noting that most microorganisms isolated from BRONJ lesions are sensitive to penicillin, making oral amoxicillin (1.5–3 g daily) and amoxicillin with a beta-lactamase inhibitor such as clavulanic acid common treatment options [35]. For patients allergic to penicillin, or in cases of antibiotic resistance, alternatives such as fluoroquinolones, metronidazole, clindamycin, doxycycline, and erythromycin can be used [74]. Some studies recommend fluoroquinolones, such as moxifloxacin or ciprofloxacin, for the empirical treatment of stage 2 or 3 BRONJ [35]. However, it is important to exercise caution when administering these drugs as they can have severe side effects in elderly and multiply ill patients.

Despite the importance of antibiotic use, studies using 16S rDNA molecular techniques have shown no significant difference in terms of bacterial diversity between the BRONJ tissue samples of patients receiving systemic antibiotics and those not receiving them. According to research, systemic antibiotics may not effectively limit bacterial colonization after the onset of BRONJ [5]. Furthermore, studies indicate that oral antibiotics have limited impact on the bacterial populations colonizing exposed bone sites in patients with BRONJ [17].

**Figure 2:**
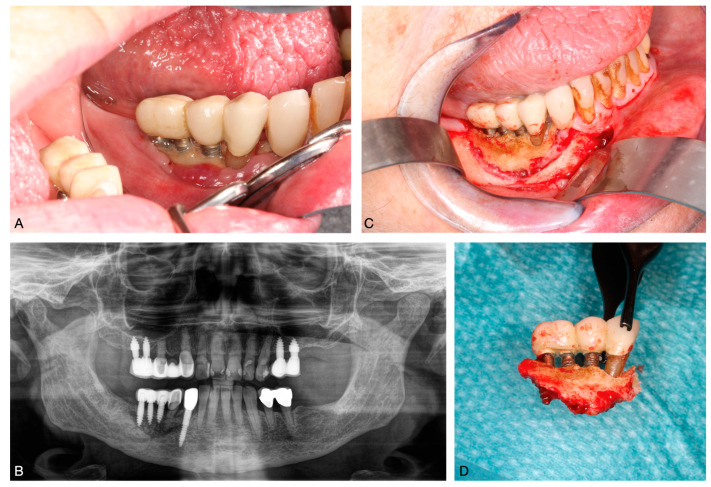
BRONJ of the lower jaw following implant placement. (**A**): exposed bone in the oral cavity; (**B**): panoramic radiograph showing a bone sequester; (**C**): intraoperative exposure of the sequester; (**D**): removed bone sequester.

Stage 3 BRONJ is associated with severe complications such as exposed and necrotic bone; invasive fistula that extend beyond the alveolar bone area, resulting in pathological fractures; extraoral fistula; oral antral or oral nasal communication; or osteolysis [6]. In these advanced cases, aggressive surgical procedures such as extensive debridement or resection may be necessary [5,75] (Figure 2).

In the evolving treatment landscape of BRONJ, researchers are investigating various non-surgical and pharmaceutical approaches in order to improve success. Teriparatide, a type of parathyroid hormone, has shown promise in promoting bone healing in BRONJ in both animal studies and limited human studies [5]. In addition, in empirical research, the effectiveness of using pentoxifylline and vitamin E together to treat BRONJ lesions has been demonstrated [5,76]. Pentoxifylline is a methylxanthine derivative that improves blood flow and reduces inflammation by increasing red blood cell deformability, reducing blood viscosity, and promoting capillary dilation. Tocopherol, also known as vitamin E, is an antioxidant that protects cell membranes and reduces tissue fibrosis. The immunomodulatory effects of pentoxifylline help to reduce inflammation, while tocopherol’s antioxidant properties protect tissues from further damage. The combined effects of these compounds facilitate bone healing and reduce inflammation in conditions such as BRONJ, osteoradionecrosis (ORN), and chronic osteomyelitis [76].

Another potential treatment strategy is discontinuing medication. Research has shown that discontinuing zoledronic acid for six to ten weeks does not affect the severity or incidence of BRONJ, indicating a long-lasting effect on bone tissue. In contrast, discontinuing RANKL inhibitors can result in at least a partial reversal of ONJ symptoms and a reduction in osteonecrotic areas and bone strain. These findings suggest that discontinuing denosumab may be more effective than BP treatment in alleviating ONJ [5]. This highlights the importance of tailored treatment strategies in managing ONJ, considering the specific medication and its impact on bone health.

## 6. Conclusions

This review critically describes the pathophysiology of BRONJ, outlining clinical risk factors and treatment options. While the causal pathophysiological mechanisms remain unknown, recent studies found a significant correlation between microbial colonization and BRONJ progression. Risk factors have been identified, such as tooth extraction, dentoalveolar surgery, high BP dosages, and specific patient demographics, contributing to a better understanding of disease susceptibility. Current treatment approaches include conservative and surgical measures and depend on disease stage, with an increasing emphasis on understanding microbial dynamics and dysbiosis for treatment guidance. Despite recent progress, finding comprehensive, multidisciplinary treatment strategies remains a crucial but challenging task, given the complex nature of BRONJ and its impact on patients’ quality of life. Future research should focus on elucidating the interplay between BPs, the oral microbiome, and the immune response to develop targeted therapies that lead to risk reduction and outcome improvements for patients suffering from BRONJ.

## Data Availability

Not applicable.
